# Validation of a Novel Method for Measuring Sweat-Derived Immunoglobulin A

**DOI:** 10.7759/cureus.100811

**Published:** 2026-01-05

**Authors:** Sayuri Goryoda, Mio Yamasawa, Yuho Ogasawara, Hyugo Tsukada, Syunsuke Ueda, Mai Wako, Kuniaki Nagamine

**Affiliations:** 1 Integrated Food and Agricultural Science, Yamagata University, Tsuruoka, JPN; 2 Agri-Food Branding, Yamagata University Advanced Agri-Food System Research Center (YAAS), Tsuruoka, JPN; 3 Behavioral Science, Well-Being Institute, Yamagata University, Yamagata, JPN; 4 Graduate School of Organic Materials Science, Yamagata University, Yonezawa, JPN; 5 Polymeric and Organic Materials Engineering, Yamagata University, Yonezawa, JPN

**Keywords:** iga, immune system, saliva, skin surface, sweat

## Abstract

Introduction: Immunoglobulin A (IgA) is a major antibody responsible for mucosal immunity, abundant in saliva, tears, airways, and the gastrointestinal tract, where it functions as the first line of defense against pathogens. In recent years, simple and non-invasive methods for monitoring IgA levels have become increasingly important for health management and disease prevention. Blood and saliva are routinely used to detect IgA levels; however, blood sampling is invasive, and saliva is strongly influenced by psychological states, limiting high-frequency or real-time monitoring. This study evaluated the feasibility of measuring IgA from sweat using a non-invasive skin surface collection method.

Methods: The study included 60 non-medical undergraduate and graduate students (25 males, 35 females; mean age 20.5 ± 1.4 years). Participants with skin disorders, immune dysfunctions, or active infections were excluded. Sweat samples were collected during three periods in 2023 (June, July-August, November) in a laboratory setting maintained at 18-25°C. After hand washing and disinfection, 100 µL of phosphate-buffered saline (PBS) was applied to the fingertip with a cap fixed by medical tape and exposed for one, three, five, or 30 min. PBS was recovered, frozen at -20°C, and analyzed for secretory IgA (s-IgA) using enzyme-linked immunosorbent assays (ELISA). Two commercial kits (Yanaihara Institute and Abcam) were used, with all assays conducted by the same investigator. Short exposure conditions (1-5 min) yielded IgA below the detection threshold. However, under the 30-minute exposure condition, IgA was successfully detected. In the third collection period using the Abcam kit, quantification success rates were 83%, 90%, and 90% across three repeated measurements.

Results: Mean IgA concentrations were 5.37 ± 11.27, 5.81 ± 10.10, and 7.96 ± 12.47 ng/mL. Although some samples exceeded the upper or lower detection limits, reproducibility was acceptable, showing that sweat IgA can be reliably measured. To the best of our knowledge, this study is the first to demonstrate successful detection of sweat IgA using a simple, non-invasive PBS exposure method. Compared with blood or saliva testing, this technique is less invasive and suitable for routine use. Integration with biosensing and wearable technologies may enable real-time monitoring of immune function in daily life. Challenges remain, including individual variability, environmental influences, and standardization of procedures. Future research should focus on the following objectives: increasing reproducibility, clarifying the role of lifestyle and gut microbiota in sweat IgA dynamics, and reducing the collection time for practical application.

Conclusion: This study presents a novel method for non-invasive measurement of IgA from sweat, providing a technological foundation for applications in health monitoring and preventive medicine. With further refinement, sweat IgA monitoring could become a valuable tool for routine immune assessment, early disease detection, and personalized medical care.

## Introduction

Immunoglobulin A (IgA) is an antibody that is primarily responsible for mucosal immunity and is abundant in saliva, tears, airways, gastrointestinal tract, and other secretions, where it functions as a first-line defense mechanism against pathogen invasion [1,2]. IgA is also known to play diverse roles in the mucosal immune system, including inhibition of bacterial adhesion, neutralization of viruses and toxins, and prevention of antigen absorption [3,4]. Secretory IgA (s-IgA) is the most abundant antibody produced in mice and humans and is predominantly distributed in mucosal tissues, particularly in the intestinal tract [5,6]. It plays an important role in neutralizing pathogens, especially in the early stages of infection, and plays a central role in mucosal immunity [3]. In recent years, IgA has attracted attention as a biomarker for systemic immune status [7,8] and has been shown to be affected by mental and physical stress [9]. Therefore, the development of simple immune monitoring methods using IgA is becoming increasingly important for health management and disease prevention [10].

Currently, methods for measuring IgA using blood and saliva have been developed and are being applied to stress assessment and simple testing of immune function [7,8]. However, blood sample collection is invasive and unsuitable for routine monitoring. Additionally, saliva samples are easily influenced by the patient’s psychological and physiological states, such as anxiety and tension, as well as diurnal variation, fatigue, physical activity, sleep deprivation, and hormonal status, which limits stable real-time and high-frequency evaluation of immune status [11,12]. Hence, the development of non-invasive sensor technologies for biomarker detection has been progressing in recent years. Wearable devices using microfluidic and electrochemical techniques have attracted attention as a means of detecting components of sweat and saliva in real time and with high sensitivity [13,14]. Additionally, sensor technologies using flexible materials that can be directly attached to the skin are being developed for practical use [15]. However, the development of noninvasive sensor technologies for biomarker detection is still ongoing. Attempts to detect IgA from the skin surface are still limited [16,17], and there are only a few reports on the efficacy of this method. In this study, conducted between June and November 2023, we aimed to investigate a non-invasive method to collect and detect sweat IgA by exposing the skin surface to phosphate-buffered solution (PBS) for a certain period under controlled laboratory conditions.

## Materials and methods

Study participants

The study participants were 60 non-medical undergraduate and graduate students (25 males and 35 females) living in a local city. The mean age of the participants was 20.5 ± 1.4 years. Those with a history of skin diseases, immune system disorders, or allergic diseases, as well as those with fever or infectious disease symptoms on the day of sample collection, were excluded.

**Figure 1:**
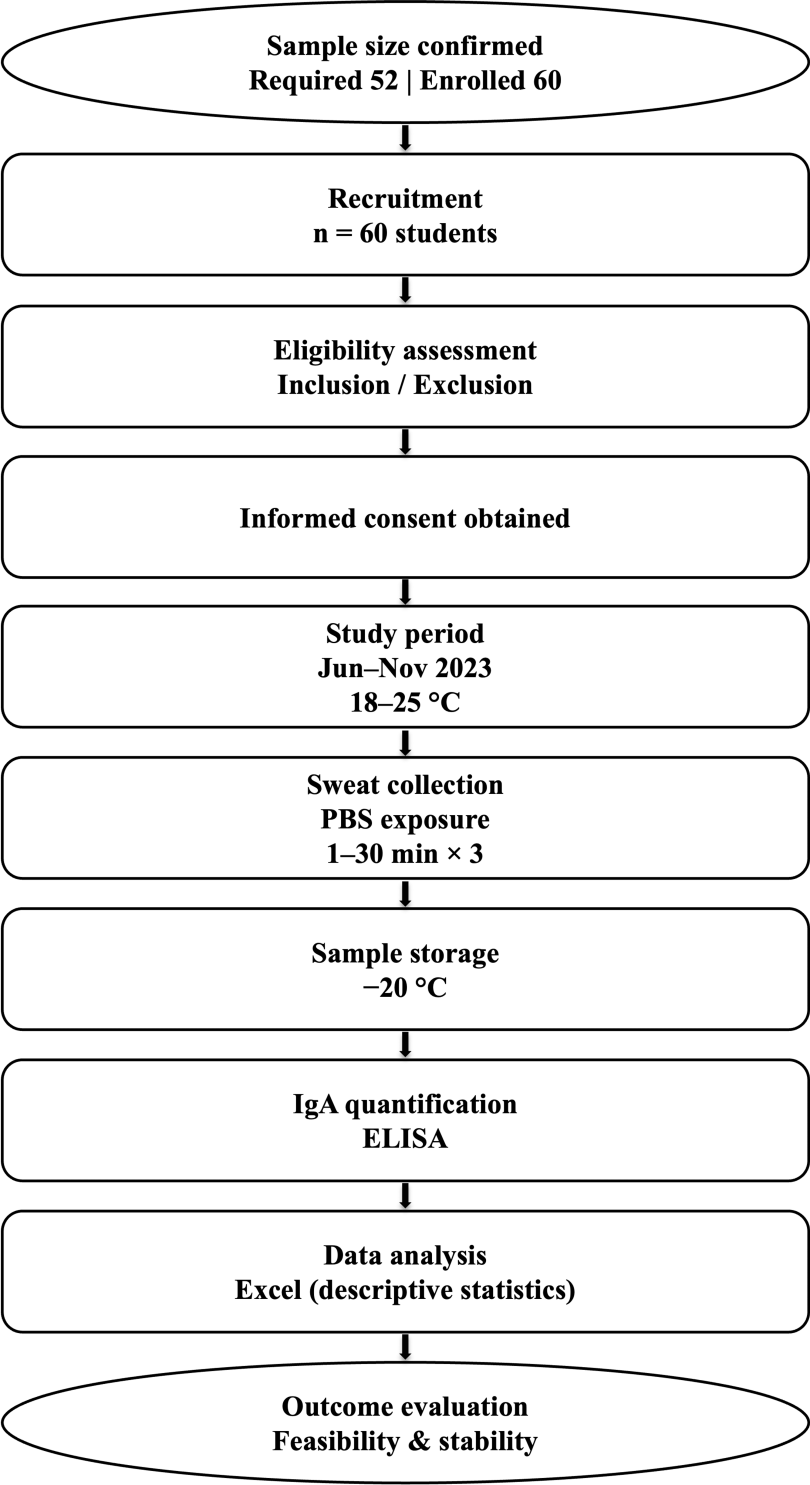
Flow chart of the study protocol IgA: immunoglobulin A; ELISA: enzyme-linked immunosorbent assay; PBS: phosphate-buffered saline

Sixty students were recruited and assessed for eligibility. As shown in Figure 1, after obtaining informed consent, the final sample size exceeded the required number. Sweat samples were collected under controlled temperature conditions and analyzed for IgA concentration using enzyme-linked immunosorbent assays (ELISA). Descriptive data analysis was performed, and the feasibility and stability of the measurement protocol were evaluated.

Sample size setting

Assuming an effect size of 0.5, a power of 0.90, and α = 0.05, the required sample size was calculated using G*Power 3.1 (Heinrich Heine University Düsseldorf, Düsseldorf, Germany) [18]. This resulted in a minimum required sample size of 52 participants, and a total of 60 participants were enrolled, exceeding this requirement.

Sample collection period and location

Sweat samples were collected in a laboratory on campus over the following three periods: June 28-30, 2023 (Cool 1), July 31-August 4 (Cool 2), and November 13-17 (Cool 3). During each period, a total of three sweat samples were collected. Measurements were performed in an environment controlled at a room temperature of 18-25°C.

Sample collection procedure and assessments

Before starting the measurement, participants washed their fingertips with soap. Afterward, they washed their index fingers with a 1 vol% ethanol solution for 15 seconds. The finger was wiped with Kimwipe and allowed to air-dry for five minutes. After drying, a cap filled with 100 µL of PBS was fixed to the tip of the index finger with medical tape and exposed to the skin surface for one minute, three minutes, five minutes, and 30 minutes. Afterward, sweat samples were collected in microtubes and frozen at -20°C. Skin surface s-IgA extracted in PBS was quantified using ELISA. The kits used were the YK280 Human s-IgA (Saliva) ELISA kit (Yanaihara Institute, Shizuoka, Japan) [19] and s-IgA ELISA kit (ab196263, Abcam, Cambridge, UK) [20], and measurements were performed according to the manufacturer’s instructions for each product. All measurements were performed by the same investigator (Table 1).

**Table 1:** Evaluation tools, scales, and measurement systems used IgA: immunoglobulin A; ELISA: enzyme-linked immunosorbent assay

Tool/Equipment/Assay	Purpose	Free to Use	License/Access Status
G*Power 3.1 [18]	Sample size calculation	Yes	Free and open-source software
YK280 Human s-IgA ELISA kit [19]	Quantification of secretory IgA	No	Commercially available kit; open-use for research per manufacturer’s instructions
Abcam Human s-IgA ELISA kit [20]	Quantification of secretory IgA	No	Commercially available kit; open-use for research per manufacturer’s instructions
Thermo Scientific Multiskan SkyHigh Microplate Reader	Absorbance measurement for ELISA	No	Commercial laboratory equipment; no license required

Statistical analysis

Statistical analyses were performed using Microsoft Excel version 365 (Microsoft Corporation, Redmond, WA, USA).

Equipment used

A Thermo Scientific Multiskan SkyHigh microplate reader (Thermo Fisher Scientific, USA) was used for absorbance measurements. A -20°C freezer was used for cryopreservation.

Ethical considerations

Participants were informed of the purpose and procedures of the study in advance, and informed consent was obtained in writing. After obtaining signatures on consent forms, all data were anonymized and managed with de-identified numbers. The conduct of this study complied with the Declaration of Helsinki. The study was reviewed and approved by the Ethics Committee of Yamagata University School of Medicine (approval number: 2022-335).

## Results

During the first course of sample collection and assessments (June 28-30, 2023), ELISA kits manufactured by Yanaihara Institute were used for all exposure conditions (one, three, and five minutes). IgA was undetectable in the sweat samples collected from the fingertips because the levels were below the detection threshold of the kit (≤82 ng/mL). During the second course of sample collection and assessments (July 31-August 4, 2023), under the 30-minute exposure condition, IgA was undetectable with the kit manufactured by Yanaihara Institute, because the levels were below the detection threshold of the kit, as in the first course.

During the third course of sample collection and assessments (November 13-17, 2023), all three quantifications were performed using the Abcam kit under 30-minute exposure conditions, and the results are reported as follows (Tables 2-3).

**Table 2:** Sweat IgA collected after exposing the skin surface to PBS for 30 minutes IgA: immunoglobulin A; PBS: phosphate-buffered saline

Sweat	First	Second	Third
Total sample size	60	60	60
Above the upper limit	5	3	5
Below the lower limit	5	3	1
Percentage of quantitative success	83	90	90
Average IgA (ng/mL)	5.37 ± 11.27	5.81 ± 10.10	7.96 ± 12.47

**Table 3:** IgA collected from saliva IgA: immunoglobulin A

Saliva	First	Second	Third
Total sample size	60	60	60
Above the upper limit	5	3	1
Below the lower limit	0	0	0
Percentage of quantitative success	92	95	98
Average IgA (µg/mL)	218.19 ± 207.98	191.99 ± 178.91	270.89 ± 330.88

During the first measurement (quantification success rate: 83%), of the 60 samples, five had values that were above the upper limit of the measurement range, and five had values that were below the lower limit of the measurement range. During the second measurement (quantification success rate: 90%), of the 60 samples, three had values that were above the upper limit of the measurement range, and three had values that were below the lower limit of the measurement range. During the third measurement (quantification success rate: 90%), of 60 samples, five had values that were above the upper limit of the measurement range, and one had a value that was below the lower limit of the measurement range. The average sweat IgA concentrations were 5.37 ± 11.27, 5.81 ± 10.10, and 7.96 ± 12.47 ng/mL for the first, second, and third measurements, respectively.

## Discussion

In this study, sweat IgA was successfully detected for the first time using a non-invasive collection method involving skin-surface exposure to PBS. Traditionally, IgA has been mainly measured using body fluids, including blood and saliva; however, there were concerns regarding the invasiveness and stability of the samples [7,9]. Although blood-based measurements are highly accurate, they are unsuitable for routine health monitoring due to challenges associated with sample collection. In this study, we focused on sweat, which has not been sufficiently investigated as a source for IgA measurement, and were able to establish a simpler and non-invasive method for IgA measurement. This approach offers practical improvements over conventional methods. To the best of our knowledge, this is the first study to demonstrate the feasibility of detecting IgA non-invasively from the skin surface, representing a novel biological measurement compartment distinct from conventional serum- or saliva-based assessments. This conceptual shift expands the scope of immunological monitoring beyond traditional body fluids.

We further confirmed that even with a short skin exposure time of 30 minutes, quantifiable levels of IgA could be stably recovered. This may be because the procedure involved adhering PBS to the fingertips with medical tape, which created conditions for efficient recovery of sweat IgA at relatively high concentrations. The results provide insights for further optimization of non-invasive measurement techniques, and the reduction of measurement time is an important factor that could promote practical application. Importantly, the non-invasive nature of the skin-based approach allows repeated measurements without physical or psychological burden on participants. In the present study, IgA was measured across three repeated attempts, enabling the assessment of measurement stability and within-individual reproducibility. This repeated-measurement design strengthens confidence in the feasibility and robustness of the proposed method.

By integrating technologies and knowledge from different disciplines, this study was able to establish a new approach and obtain findings that are difficult to achieve with conventional biological detection methods. In particular, the fusion of biosensing and biomolecular detection technologies has attracted increasing attention in recent years, enabling improved detection sensitivity for trace constituents that have been difficult to detect using conventional measurement methods. The successful development of a new measurement method through the integration of knowledge from different fields is a major achievement of this research and has great potential for future research and development. This could also aid the development of bio-component measurement technology through the integration of knowledge from public health and engineering. In addition, the simplicity and practicality of the proposed measurement procedure suggest its potential applicability in real-world, community-based, and field settings, where conventional laboratory-based assays such as ELISA may be impractical.

Furthermore, with the recent developments in healthcare technology, assessment of IgA in sweat in real-time may be achievable in the future [21]. Although prior studies have been conducted on the practical application of sweat-based biometric measurements [22], the measurement of IgA in this study is novel and lays the groundwork for future research and practical applications [23]. Once this technology is fully developed, it holds promise for wide applicability, including monitoring of individual health status, early disease detection, and even application to personalized medicine.

Despite the success of this study, some limitations need to be addressed. Variations and reproducibility of measured values are important issues that need to be considered. Slight differences in sweating volume, skin condition, collection method, and other factors may affect the results of IgA concentration measurements; hence, standardization procedures must be established to minimize measurement error. Verification of reproducibility under diverse environmental conditions and improvement of the reliability of this approach are essential for future technological development. In addition, although ELISA is the established gold standard for IgA quantification and is widely used in diagnostic accuracy studies, the present study did not aim to evaluate the diagnostic performance of skin-based IgA measurement in terms of sensitivity and specificity relative to serum or salivary IgA. ELISA was employed for IgA quantification; however, it was not applied as a reference standard for formal diagnostic validation. This is because the primary objective of this study was to explore the feasibility of non-invasive IgA detection from the skin surface as a distinct biological compartment, rather than to classify individuals based on systemic IgA status. Accordingly, sensitivity and specificity analyses were beyond the scope of this exploratory, proof-of-concept study, and this limitation has been explicitly acknowledged. Moreover, this study did not sufficiently examine individual differences in sweat IgA concentrations among participants. Future studies should consider evaluating the effects of participants’ ages and lifestyle factors on IgA measurements from sweat [24,25]. IgA secretion is related to the intestinal microflora [26-28]; hence, it is also important to elucidate how the intestinal environment influences the dynamics of IgA in sweat. The composition of the gut microbiota is known to be closely related to diet, stress, and environmental factors, all of which affect the production of IgA [29,30]. Therefore, analyzing the relationship between changes in the gut microbiota and sweat IgA would allow for a more comprehensive immune monitoring system.

Addressing these issues will aid the implementation of the sweat IgA measurement technology established in this study. This will also promote its use as a new indicator for health management and disease prevention. Its widespread adoption as a system for easy and routine assessment of immune status could establish it as a standard evaluation approach in health checkups and medical care settings. Furthermore, the integration of this technology with wearable devices will promote real-time immune monitoring, contributing to the development of preventive medicine and personalized medical care. We strongly hope that the results of this research will enable the implementation of sweat IgA assessment as a new standard for evaluating immune function, contributing to the creation of a safer and more comfortable society through innovations in science and technology.

## Conclusions

In this study, sweat IgA was successfully detected for the first time with a non-invasive method involving exposure of the skin surface to PBS. These findings provide a technological framework for further research and practical application. This new sweat IgA assessment approach is noteworthy and enables simple and rapid evaluation of biological components. The findings obtained in this study are also likely to contribute to the development of simplified evaluation methods for immune function and the advancement of applied technologies for health management and disease prevention. In the future, it is expected that a more versatile system will be developed through improvements in measurement conditions and equipment.

## References

[1] Strugnell RA, Wijburg OLC (2010). The role of secretory antibodies in infection immunity. Nat Rev Microbiol.

[2] Woof JM, Russell MW (2011). Structure and function relationships in IgA. Mucosal Immunol.

[3] Abdul-Kareem HH, Al-Maqtoofi MY, Burghal AA (2023). Impact of COVID-19 vaccination on saliva immune barriers: IgA, lysozyme, and lactoferrin. Arch Virol.

[4] Scheurer S, Junker AC, He C, Schülke S, Toda M (2023). The role of IgA in the manifestation and prevention of allergic immune responses. Curr Allergy Asthma Rep.

[5] Di Renzo L, Franza L, Monsignore D (2022). Vaccines, microbiota and immunonutrition: food for thought. Vaccines (Basel).

[6] Calder PC (2013). Feeding the immune system. Proc Nutr Soc.

[7] Nagakubo D, Kaibori Y (2023). Oral microbiota: the influences and interactions of saliva, IgA, and dietary factors in health and disease. Microorganisms.

[8] Ahmadiafshar A, Mohsenifard MR, Mazloomzadeh S (2015). Evaluation of serum & salivary IgA in patients with type 1 diabetes. PLoS One.

[9] Khalid W, Arshad MS, Jabeen A, Muhammad Anjum F, Qaisrani TB, Suleria HA (2022). Fiber-enriched botanicals: a therapeutic tool against certain metabolic ailments. Food Sci Nutr.

[10] Wu Y, Wan J, Choe U (2019). Interactions between food and gut microbiota: impact on human health. Annu Rev Food Sci Technol.

[11] Engeland CG, Hugo FN, Hilgert JB (2016). Psychological distress and salivary secretory immunity. Brain Behav Immun.

[12] Seizer L, Stasielowicz L, Löchner J (2024). Timing matters: a meta-analysis on the dynamic effect of stress on salivary immunoglobulin. Brain Behav Immun.

[13] Mohan AMV, Rajendran V, Mishra RK, Jayaraman M (2020). Recent advances and perspectives in sweat based wearable electrochemical sensors. TrAC Trends Anal Chem.

[14] Nagamine K, Tokito S (2021). Organic-transistor-based biosensors interfaced with human skin for non-invasive perspiration analysis. Sens Actuators B Chem.

[15] Saha T, Del Caño R, De la Paz E, Sandhu SS, Wang J (2023). Access and management of sweat for non-invasive biomarker monitoring: a comprehensive review. Small.

[16] Imayama S, Shimozono Y, Hoashi M, Yasumoto S, Ohta S, Yoneyama K, Hori Y (1994). Reduced secretion of IgA to skin surface of patients with atopic dermatitis. J Allergy Clin Immunol.

[17] Katchman BA, Zhu M, Blain Christen J, Anderson KS (2018). Eccrine sweat as a biofluid for profiling immune biomarkers. Proteomics Clin Appl.

[18] Faul F, Erdfelder E, Lang AG, Buchner A (2007). G*Power 3: a flexible statistical power analysis program for the social, behavioral, and biomedical sciences. Behav Res Methods.

[19] Yanaihara Laboratories, “YK280 “YK280 (2024). s-IgA: Human s-IgA (Saliva) ELISA Kit (YK280). Manual.

[20] (2024). ab196263 - human IgA SimpleStep ELISA® kit. https://www.abcam.co.jp/products/elisa-kits/human-iga-elisa-kit-ab196263.

[21] Hayashi H, Sakamoto N, Hideshima S (2020). Tetrameric jacalin as a receptor for field effect transistor biosensor to detect secretory IgA in human sweat. J Electroanal Chem.

[22] Ohashi K, Osaka T (2023). Skin-attached biosensor. J Surf Finish Soc Jpn.

[23] Tsukada H, Wako M, Ueda S, Nagamine K (2024). Touchpad-based immunochromatographic strip for detecting the skin surface proteins. Anal Biochem.

[24] Saiga A, Kunisawa J (2018). Immune regulation through the "food-gut bacteria-host" network via edible oils [Article in Japanese]. Jpn J Intest Microbiol.

[25] Shete MD, Patil DB, Karade P, Chopade R, Gandhi N, Alane U (2021). Assessment of age-related changes of salivary immunoglobulin A levels among healthy individuals. J Pharm Bioallied Sci.

[26] Yoshii T, Hosomi K, Kunisawa J (2022). Regulation of immune function via metabolites of intestinal bacteria [Article in Japanese]. Jpn J Intest Microbiol.

[27] Pabst O, Slack E (2020). IgA and the intestinal microbiota: the importance of being specific. Mucosal Immunol.

[28] Massacand JC, Kaiser P, Ernst B, Tardivel A, Bürki K, Schneider P, Harris NL (2008). Intestinal bacteria condition dendritic cells to promote IgA production. PLoS One.

[29] Hosomi K, Kunisawa J (2023). The relationship between food and intestinal bacteria in the construction of the intestinal metabolic environment and its impact on health [Article in Japanese]. J Jpn Biochem Soc.

[30] Nakajima A, Sasaki T, Itoh K (2020). A soluble fiber diet increases Bacteroides fragilis group abundance and immunoglobulin a production in the gut. Appl Environ Microbiol.

